# The Clinical Significance of DNA Damage Repair Signatures in Clear Cell Renal Cell Carcinoma

**DOI:** 10.3389/fgene.2020.593039

**Published:** 2021-01-08

**Authors:** Ergang Guo, Cheng Wu, Jun Ming, Wei Zhang, Linli Zhang, Guoqing Hu

**Affiliations:** Department of Oncology, Tongji Hospital, Tongji Medical College, Huazhong University of Science and Technology, Hubei, Wuhan

**Keywords:** DNA damage, DNA repair, clear cell renal cell carcinoma, urology, immunotherapy, cancer

## Abstract

DNA damage repair plays an important role in cancer’s initiation and progression, and in therapeutic resistance. The prognostic potential of damage repair indicators was studied in the case of clear cell renal cell carcinoma (ccRCC). Gene expression profiles of the disease were downloaded from cancer genome databases and gene ontology was applied to the DNA repair-related genes. Twenty-six differentially expressed DNA repair genes were identified, and regression analysis was used to identify those with prognostic potential and to construct a risk model. The model accurately predicted patient outcomes and distinguished among patients with different expression levels of immune evasion genes. The data indicate that DNA repair genes can be valuable for predicting the progression of clear cell renal cell carcinoma and the clinical benefits of immunotherapy.

## Introduction

Renal cell carcinoma (RCC) is a lethal cancer of the urinary system, accounting for 2–3% of all malignant cancers in adults (Siegel et al., 2019). Although surgical and systemic therapies for clear cell renal cell carcinoma (ccRCC), the most common subtype of renal carcinoma, have improved survival, the outcome in patients with advanced, metastatic disease is still poor (Garje et al., 2020). Risk-stratifying the patients based on their clinical characteristics and then individualizing treatment according to the risk level could be helpful to improve the outcome. However, the current tumor stage system is insufficient to predict ccRCC prognosis effectively (Suh et al., 2020). Therefore, it is necessary to explore new biomarkers for prognostic prediction in patients with ccRCC.

The advances in genomics and bioinformatics in recent years have enabled the discovery of novel targets and biomarkers. Many biomarkers including lncRNA, miRNA, and mRNA have been identified for making diagnosis and predicting prognosis as well as guiding treatment choices (Wang et al., 2016; Carril-Ajuria et al., 2019). For instance, some immune-associated signatures have been employed to evaluate the tumor microenvironment (TME) infiltration characterization, revealing a linkage between the TME and clinical features (Zeng et al., 2019). Moreover, the signatures such as hypoxia, N6-methyladenosine (m6A) mRNA modification, autophagy and metabolism have been used for prognosis prediction (Feng et al., 2020; Hu et al., 2020; Jiang p. et al., 2020; Liu et al., 2020).

The role of DNA damage repair (DDR) in neoplasia and tumor progression has been extensively studied (Mauri et al., 2020). Defective DDR can lead to accumulated DNA lesions and genome instability, which contribute to tumorigenesis. It has been reported that germline mutations in exonuclease 5 can impair DNA repair ability and cause androgen-related prostate cancer (Ali et al., 2020). However, during cancer progression, DNA repair may be related to sensitivity to anticancer drugs such as poly ADP-ribose polymerase (PARP) inhibitors or to radiation. MAP kinase-ERK kinase5 (MEK5) can promote the phosphorylation of DNA-PK in response to ionizing radiation (Broustas et al., 2020). High TTK protein kinase (TTK) expression in breast cancer is associated with efficient repair through homologous recombination and low radiation sensitivity (Chandler et al., 2020). These earlier studies point to the importance of exploring the different roles of DNA repair genes in cancer.

As an aggressive tumor, RCC is characterized by high genomic mutation levels (Alexandrov et al., 2013; Cancer Genome Atlas Research Network, 2013; Ged et al., 2020). Many studies reported the prognostic and biological significance of the cancer-driven genetic alterations including von Hippel-Lindau, TP53 as well as PTEN mutations in RCC (Chen et al., 2019; Huang et al., 2020). However, the role of DNA repair genes in maintaining genome stability in RCC is rarely reported.

In this study, we collected and analyzed data from the International Cancer Gene Consortium, the Gene Expression Omnibus (GEO) and The Cancer Genome Atlas (TCGA) to determine which DNA repair genes are prognostic for patients with ccRCC, developed a prediction model based on the expression of DDR-associated genes, and explored genes and pathways associated with the gene signatures.

## Materials and Methods

### Data

In total, 546 DDR-associated genes were analyzed in terms of gene ontology (GO). The clinical data and RNA sequences were downloaded from the TCGA portal, which contains 514 tumor and 72 normal samples. Pathological clinical features for each patient are presented in Table 1. The other three profiles were collected from the GEO (GSE17818 and GSE53757) and ICGC databases.

**TABLE 1:** Clinical characteristics of the ccRCC patients.

	E		High risk group (251)		Low risk group (263)	
	Number	Percentage (%)	Number	Percentage (%)	Number	Percentage (%)
**Age**						
>60	273	53.1	141	56.2	132	50.2
≤60	241	46.9	110	43.8	131	49.8
**Gender**						
Female	178	34.6	80	31.9	98	41.5
Male	336	65.4	171	68.1	165	58.5
**Grade**						
G1	13	2.5	1	0.4	12	4.6
G2	224	43.6	89	35.5	135	51.3
G3	204	39.7	102	40.7	102	38.8
G4	73	14.2	59	23.4	14	5.3
**Stage**						
I	257	50	96	38.2	161	61.2
II	53	10.3	22	8.8	31	11.8
III	122	23.7	70	27.9	52	19.8
IV	82	26	63	25.1	19	7.2

### Screening for Differentially Expressed Genes

The original data were organized and analyzed with the help of R software (4.0.1). Genes that showed a log_2_ fold change >1 and a false discovery rate <0.01 between tumor and normal tissues were considered to be differentially expressed. Venn diagrams were used to determine the interaction of the differentially expressed genes (DEGs) of the four datasets. The differentially expressed DDR genes between tumor and normal tissues was identified using the “Linear Models for Microarray Data (LIMMA)” package with a cut-off criterion of *p* < 0.05.

### Modeling and Assessment of Its Prognostic Ability

The patients in the TCGA database were allocated randomly into one of two groups: a training group and a validation group. The training group was used to construct a clinical prognostic model, and the validation group was used to evaluate the model’s stability. First, univariate Cox regression analysis was used to extract potential DNA repair genes significantly related to patient prognosis. Last absolute shrinkage and selection operator (LASSO) regression was then used to prevent overfitting of the model. Finally, a risk score formula was generated by combining the gene expression levels weighted by the regression coefficients derived from the multivariate Cox regression analysis.

According to the risk scores calculated using the formula, we set the median risk scores as cut-off values and allocated the patients in both training and validation groups into a high-risk group and a low-risk group. The survival rates of the two groups were then compared using Kaplan-Meier analysis. The predicting sensitivity of the selected gene sets was assessed by plotting the receiver operating characteristics (ROC) curve. An area under the curve (AUC) >0.60 was taken as indicating moderate accuracy, and an AUC >0.75 was regarded as highly accurate for predictions. The prognostic values of the risk scores were assessed by the univariate and multivariate analysis through R software.

Additionally, the correlation between the risk scores and clinicopathological features in ccRCC patients were assessed based on the data from TCGA. Then we integrated our risk scores into current staging system and evaluated the utility in stratifying the risk levels.

### Functional and Pathway Enrichment Analysis

We identified the different genes between high risk and low risk groups, and conducted GO pathway analysis with the clusterProfiler R packages to functionally annotate the DEGs in different groups.

### Statistical Analyses

The *t*-test was used for statistical comparison. Long-rank test was performed to compare the overall survival rates between different groups. The statistical analyses were completed using R software, and results were considered statistically significant when the *p* value was ≤0.05.

## Results

### Differentially Expressed Genes

**FIGURE 1:**
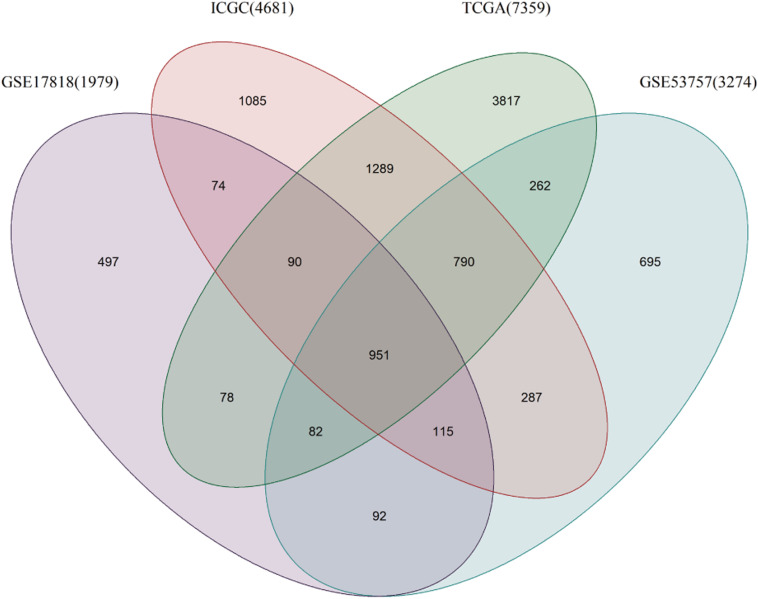
Venn diagram of genes differentially expressed between ccRCC and normal tissues.

A total of 7359 genes in the TCGA data, 2862 in the GEO data and 4681 in the ICGA data were found to be differentially expressed between the ccRCC and normal renal samples. There were 951 DEGs common to the gene lists (Figure 1), and 26 of them were found to be closely related to DDR (Table 2).

**TABLE 2:** Univariate analysis of 26 DNA damage repair genes in patients with ccRCC.

Gene name	HR	HR.95L	HR.95H	*p* value
BCL2A1	1.054477	1.023117	1.086798	0.000574
CCND1	0.997448	0.99525	0.999651	0.023176
DDB2	0.959523	0.906267	1.015908	0.15612
DDIT4	1.001353	0.999452	1.003257	0.163094
DTL	1.318731	1.062495	1.636762	0.012077
EGLN3	0.996964	0.992156	1.001796	0.217695
GINS2	1.268419	1.04468	1.540076	0.016331
HMOX1	0.998082	0.994866	1.001309	0.243801
IFI16	1.043266	1.021694	1.065293	0.0000709
ISG15	1.010463	1.005042	1.015914	0.000149
MACROD1	1.009071	0.972349	1.047181	0.633044
MNDA	1.015882	0.975147	1.058317	0.450462
MYC	1.00205	0.994208	1.009954	0.609446
NUDT1	1.176014	1.071893	1.29025	0.000609
NUPR1	1.001684	0.99866	1.004717	0.275476
E	1.021634	1.003497	1.0401	0.019187
PLK2	0.998984	0.983324	1.014894	0.899689
PYCARD	1.033763	1.012919	1.055037	0.001398
RAD51AP1	1.46565	1.23834	1.734685	0.00000874
SFRP2	1.004603	1.00157	1.007644	0.002907
SLFN11	1.063327	1.020337	1.108128	0.003544
SPATA18	0.929987	0.892106	0.969477	0.000624
STK33	1.034072	0.722815	1.479363	0.854503
TNFRSF1B	1.012184	0.991965	1.032815	0.239466
UBE2L6	1.002813	0.992091	1.013652	0.608502
VAV3	0.944322	0.904244	0.986177	0.009625

*HR abbreviates hazard ratio.*

### Prognosis-Related Genes

**FIGURE 2:**
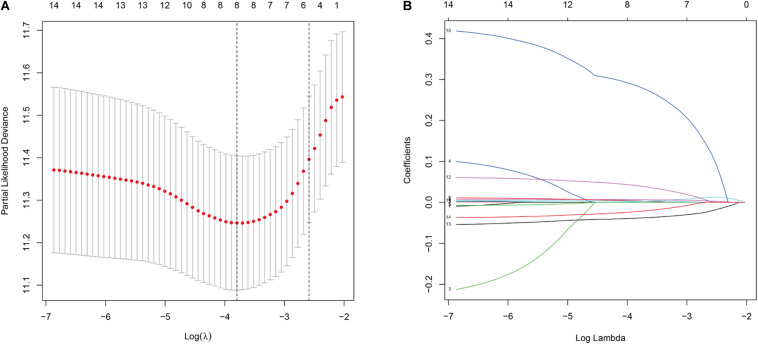
**(A)** Partial likelihood deviance versus log (λ) was performed through LASSO regression. **(B)** Coefficients of selected features are shown by lambda parameter.

Univariate analysis showed that the expression of 14 DNA repair-related genes (DRRGs) was significantly related to ccRCC patient prognosis (*p* ≤ 0.05). LASSO regression analysis then confirmed acceptable lack of collinearity among the variables (Figures 2A,B). Subsequently, multivariate Cox regression identified 6 DRRGs as potentially powerful prognostic factors (Table 3). ISG15, RAD51AP1, secreted frizzled-related protein 2 (SFRP2), and SLFN11 were considered as high-risk genes (hazard ratio >1), while SPATA18 and VAV3 were regarded as low-risk (hazard ratio <1).

**TABLE 3:** Multivariate Cox regression of 6 DNA damage repair genes and overall survival among the ccRCC patients.

Gene	Coefficient	HR	HR.95L	HR.95H	*p* value
ISG15	0.007674	1.007703	1.001845	1.013596	0.009895
RAD51AP1	0.323186	1.381522	1.141907	1.671418	0.000883
SFRP2	0.004232	1.004241	1.000589	1.007905	0.022789
SLFN11	0.056129	1.057734	1.011561	1.106014	0.013712
SPATA18	–0.04544	0.955578	0.910287	1.003123	0.06664
VAV3	–0.03492	0.965683	0.922456	1.010936	0.135047

*HR abbreviates hazard ratio.*

**FIGURE 3:**
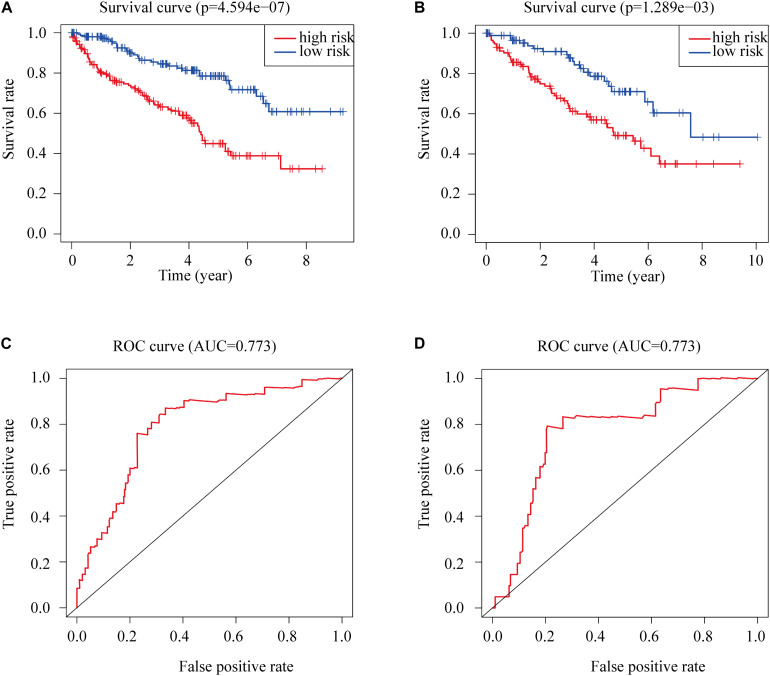
Kaplan-Meier plots of overall survival according to risk scores for **(A)** the training group and **(B)** the test group. **(C)** ROC for overall survival in the training group. **(D)** ROC for overall survival in the test group.

**FIGURE 4:**
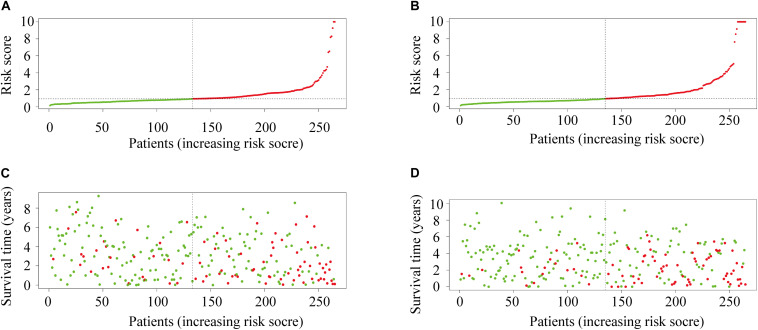
**(A)** The distribution of risk scores in the training group. **(B)** The distribution of risk score in the test group. **(C)** Survival plots of patients in the training group. **(D)** Survival plots of patients in the test group.

The Kaplan-Meier survival analysis showed that patients with high risk scores exhibited worse overall survival rates than those with low risk scores (Figures 3A,B). The ROC analysis generated an AUC of 0.791 for the training group and 0.773 for the test group, indicating good prediction performance (Figures 3C,D). The distributions of risk scores and survival statuses of the patients in the two groups are shown in Figures 4A–D.

### Risk Scores and Clinical Characteristics

**FIGURE 5:**
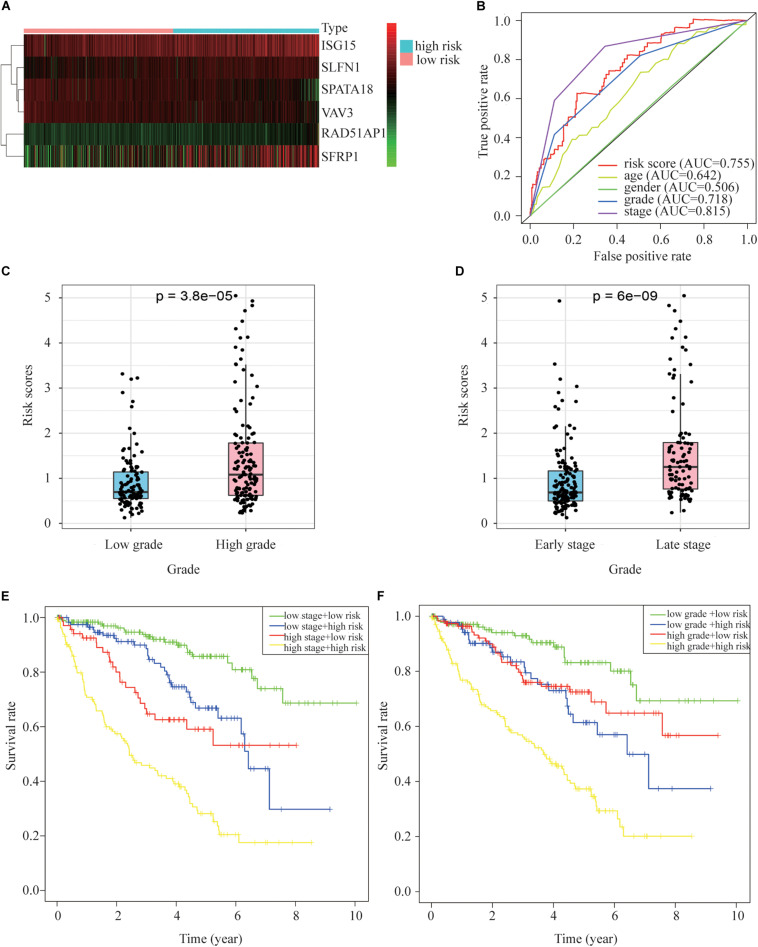
Relationships between risk scores and clinical pathology indicators. **(A)** A heat map of gene expression in the different groups. **(B)** ROC relating risk scores with clinical indicators of pathology. **(C)** Tumor grade and risk score related. **(D)** Tumor stage and risk score related. **(E)** Overall survival curve for patients with different stages and risks. **(F)** Overall survival curve for patients with different grades and risks.

The expression of prognostic genes in the two groups is presented in Figure 5A. On average, the risk scores of patients differently classified by pathological stage and WHO grade were significantly different (Figures 5A,B). The ROC curve showed that the proposed model’s predictions of overall survival were similar to those using pathological stage (Figures 5C,D). The patients in high risk group had a significantly worse prognosis than those in low risk group even though they had similar clinicopathologic characteristics, in similar stage (Figure 5E) or grade (Figure 5F).

**FIGURE 6:**
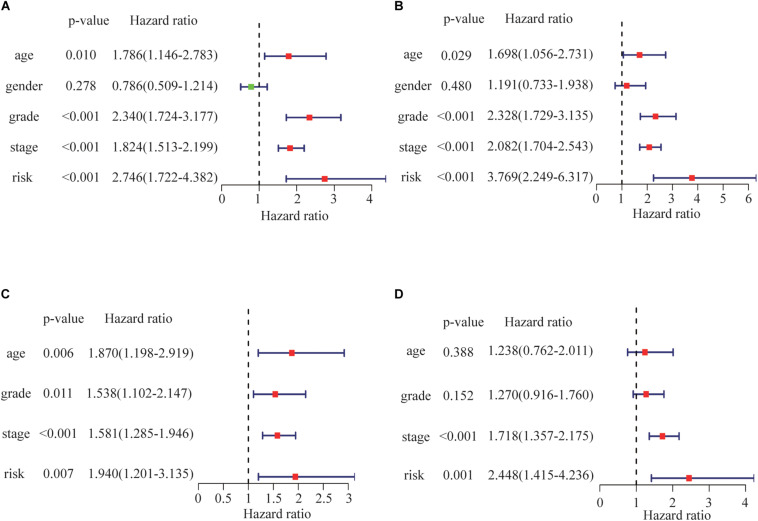
Risk score correlations with overall survival by univariate Cox regression analysis in **(A)** the training group and **(B)** the test group. Risk score as an independent prognostic indicator (by multivariate Cox regression analysis) in **(C)** the training group and **(D)** the test group.

Univariate analyses identified age, pathological stage, grade and risk score as significant predictors of OS in both the training and test groups (Figures 6A,B). Multivariate analysis confirmed that only risk score and pathological stage were related to OS independently (Figures 6C,D). Moreover, the hazard ratio of the risk is higher than other clinicopathologic characteristics, which indicating that the prognostic value of risk score based on the DDR associated genes might be better than clinical stages and grades.

### Risk Score and Immune Evasion

**FIGURE 7:**
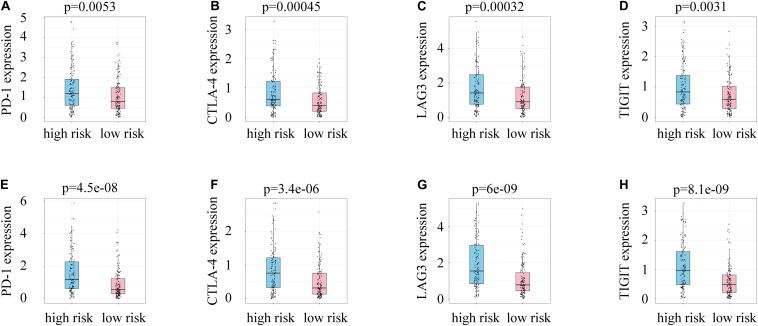
**(A)** PD-1 expression and risk scores in the training group. **(B)** CTLA-4 expression and risk scores in the training group. **(C)** LAG3 expression and risk scores in the training group. **(D)** TIGIT expression and risk scores in the training group. **(E)** PD-1 expression and risk scores in the test group. **(F)** CTLA-4 expression and risk scores in the test group. **(G)** LAG3 expression and risk scores in the test group. **(H)** TIGIT expression and risk scores in the test group.

A link between DDR and immune escape has been reported (Sato et al., 2019), so immunity-related gene expression was evaluated in patients with different risk scores. Patients in the high-risk group had higher expression of PD-1, LAG-3, CTLA-4, and TIGIT than those in the low-risk group (Figures 7A–H), indicating that patients with high risk scores might benefit from the use of combination therapies that integrate immunotherapy with chemotherapy, radiotherapy, and targeted therapy.

### Enrichment in Patients With Different Risk Scores

**FIGURE 8:**
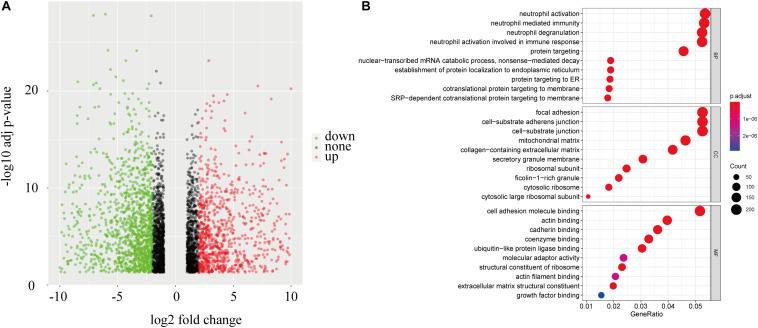
Enrichment in patients with different risks. **(A)** A volcano plot of genes in ccRCC patients with low and high risk scores. **(B)** Functional annotation of the DEGs based on gene ontology classification.

To identify a mechanism potentially contributing to tumor progression, 4190 genes differentially expressed between the high-risk and low-risk groups (Figure 8A) were studied. GO enrichment analysis showed that the DEGs are mainly related to immune processes such as neutrophil degranulation, neutrophil-mediated immunity, neutrophil activation, and in metastasis pathways including cell adhesion molecule binding, cell-substrate adherent junctions, and focal adhesion (Figure 8B). Those relate the prognostic model mechanistically to tumor progression in ccRCC patients.

## Discussion

Clear cell renal cell carcinoma is highly heterogeneous, and its prognosis can vary widely even for patients with similar clinicopathologic characteristics and treatment options, suggesting that current classifications are insufficient for assessing outcomes and risk stratification. Hence, more research is needed to identify novel biomarkers and risk factors in patients with ccRCC. The current study was performed as a pilot trial not only to identify the potential biomarkers associated with prognosis but also to explore new hypotheses for further studies.

Prediction models have for years been explored to guide individual treatment. It has been reported that models based on the expression of tumor genes would allow prediction of patients’ response to fluorouracil and gemcitabine (Clayton et al., 2020). Jiang et al.(2019; Jiang Y. Z. et al., 2020) classified triple-negative breast cancers into different subtypes on the basis of genomic features and evaluating the clinical benefit of subtyping-based targeted therapy for triple-negative breast cancers. But the individual treatment based on molecular subtyping in ccRCC is rare.

The DDR-associated pathway is associated with the carcinogenesis of ccRCC, and the expression of some DNA repair factors is correlated with patient prognosis (Na et al., 2019). This study was designed to determine the impact of DNA repair genes on the progression of ccRCC and patient prognosis. Twenty-six DNA repair genes were identified and 14 of them were considered as related to the overall survival of ccRCC patients, and risk scoring based on those genes was shown to be a useful independent prognostic technique. Patients identified as high-risk through the scoring tended to have late stage disease and high tumor grades.

RAD51-associated protein 1 (RAD51AP1) interacts with RAD51, which plays an important role in RAD51-related homologous recombination (Wang et al., 2019). A recent study found that RAD51AP1 is highly expressed in non-small cell lung cancer patients and facilitates invasion and metastasis by inducing epithelial-mesenchymal transitions (Wu et al., 2019). ISG15 is related to chemosensitivity in pancreatic cancer (Ina et al., 2010). As a known regulator of the WNT pathway, SFRP2 can activate or suppress canonical and non-canonical WNT pathways in different tissues. Sun reports that DNA damage can induce the production of SFRP2, which enhances WNT16B and β-catenin activity to result in therapy resistance (Sun Y. et al., 2016). Recent studies have found that Schlafen 1 (SLFN1) produces cells that are highly sensitive to DNA-damaging drugs by suppressing checkpoint maintenance and repair through homologous recombination (Mu et al., 2016). Small cell lung cancer patients with high SLFN1 expression might benefit from PARP inhibition (Murai et al., 2016; Lok et al., 2017).

The immunotherapy blocking the interaction between PD-1 and PD-L1 in ccRCC patients has seen advances in recent years, which provides a new treatment option (Motzer et al., 2015). However, the benefit from this therapy is highly variable (Motzer et al., 2020). Although there are some markers developed to predict immunotherapy response, their specificity remains controversial.

DNA damage repair is associated with immune activation in different cancers. Chatzinikolaou et al. (2014) has reviewed the direct links between innate immune signaling and DNA damage. And recent work has revealed that pharmacological inhibition of the DNA damage response proteins CHK1 and PARP increases the levels of tumor-infiltrating T-lymphocytes. With anti-PD-L1 therapy it has been shown to work synergistically in modes of small cell lung carcinoma through the STING/TBK1/IRF3 innate immune pathway (Sen et al., 2019). A group led by Sato has shown that genotoxic stress such as irradiation or PARP inhibition can upregulate the expression of PD-L1 through the ATM-ATR/CHK1 pathway (Sato et al., 2017). Jiao et al. (2017) found that PARP inhibitors can upregulate PD-L1 and promote immune suppression. Garsed et al. (2018) reported that DDR pathway mutations are associated with immune cell infiltration and activation. The association of mutations in DNA repair genes and immune regulatory genes with bladder cancer has also been documented (Vidotto et al., 2019). Moreover, it has been reported that alterations in DDR genes which cause loss of function are frequent in metastatic ccRCC, which may certainly affect the effectiveness of immunotherapy (Ged et al., 2020). We therefore performed this bioinformatics analysis to explore the potential relationship between DDR and immune evasion. Our study found that ccRCC patients with greater risk had high expression of immune evasion genes. As it has been reported that antibodies against immune evasion genes could restore responses of tumor-associated T cells to tumor related antigens (Dong et al., 2002), and higher PD-L1 expression on tumor cells and/or immune cells was shown to be associated with better efficacy of anti-PD1/PD-L1 immunotherapies (Dong et al., 2002; Topalian et al., 2012), we speculated that patients with high risk might benefit from immunotherapy.

The GO of the DDR genes identified many immunity- and metastasis-related pathways. As the major portion of the leukocytes in peripheral blood, neutrophils have been associated with carcinogenesis and cancer development (Galdiero et al., 2018; Mollinedo, 2019). Many studies have shown that neutrophils in the TME are related with poor prognosis (Shen et al., 2014; Zhou et al., 2016; Jungnickel et al., 2017). Moreover, the neutrophil-to-lymphocyte ratio has been identified as a risk factor with different tumors (Templeton et al., 2014; Sun W. et al., 2016; Barker et al., 2020). Neutrophils in the TME release many inflammatory factors that contribute to tumor proliferation, metastasis and immune suppression. A group led by Coffelt found that tumor-associated neutrophils (TANs) induced by IL-17 can inactivate cytotoxic T lymphocytes and promote metastasis (Coffelt et al., 2015). A previous study found that the interaction between TANs and tumor cells led to tumor shedding, which promoted tumor spreading (Wislez et al., 2007). Moreover, TANs recruit several immunoregulatory cells which inhibit anti-tumor immunity and induce T cell apoptosis by releasing TNF-α (Tecchio et al., 2013; Powell and Huttenlocher, 2016; Michaeli et al., 2017). MMP2, MMP9, VEGF, arginase, and elastase from neutrophil degranulation can also contribute to tumor progression (Caruso et al., 2010; Mishalian et al., 2013; Deryugina et al., 2014). This study has revealed that DNA repair genes are involved in immune and metastasis signaling, uncovering their effects on the initiation and development of ccRCC.

Many prognostic models have been proposed based on immunity, autophagy and glycolysis genes, and their prognostic value in different types of cancer has been evaluated (An et al., 2018; Wan et al., 2019a,b; Zhang et al., 2019). However, the prognostic utility of DNA damage genes in cancer remains controversial. This study has shown that DDR-associated signatures correlate with poor prognosis among ccRCC patients, and the ROC curves show the model’s potential utility in predicting the survival of ccRCC patients. Moreover, integrating our risk scores into current staging system lead to a more precise predictive model to further stratify patients with distinct prognosis, as shown by the survival curves plotted in this study.

It should be noted that there are also some shortcomings in our study. First, only a few Asians were included in the data used, which may lead to selection bias. Secondly, this is a bioinformatics analysis based on public databases, and experimental as well as clinical studies are required to validate these findings.

In summary, our study identified DDR signatures that could predict prognosis in ccRCC patients. The current findings show that risk scores from our model can further improve the current clinical staging system and provide more accurate prediction on outcomes. In addition, our model can predict the expression of immune evasion proteins in ccRCC patients, which might predict their response to immunotherapy. Further studies are required to validate our findings.

## Data Availability Statement

Publicly available datasets were analyzed in this study. This data can be found here: The datasets generated for this study can be found in The Cancer Genome Atlas database (https://portal.gdc.cancer.gov/), international cancer genome consortium (https://icgc.org/), and GEO (https://www.ncbi.nlm.nih.gov/geo).

## Author Contributions

EG, CW, and JM conceived the work. EG and WZ conducted the data analysis. EG and LZ wrote the manuscript. GH revised and proofread the manuscript. All authors revised the manuscript and allowed the submitted version.

## Conflict of Interest

The authors declare that the research was conducted in the absence of any commercial or financial relationships that could be construed as a potential conflict of interest.

## Abbreviations

DDR: DNA damage repair
ccRCC: clear cell renal cell carcinoma
TCGA: The Cancer Genome Atlas
GEO: Gene Expression Omnibus
ICGC: International Cancer Genome Consortium
FC: fold change
FDR: false discovery rate
DEGs: differentially expressed genes
ROC: receiver operating characteristics
DRRGs: DNA repair-related genes
GO: gene ontology
TANs: tumor-associated neutrophils
PARP: poly ADP-ribose polymerase.

## References

[B1] Alexandrov L. B. Nik-Zainal S. Wedge D. C. Aparicio S. A. Behjati S. Biankin A. V. (2013). Signatures of mutational processes in human cancer. *Nature* 500 415–421. 10.1038/nature12477 23945592 PMC3776390

[B2] Ali S. Zhang Y. Zhou M. Li H. Jin W. Zheng L. (2020). Functional deficiency of DNA repair gene EXO5 results in androgen-induced genomic instability and prostate tumorigenesis. *Oncogene* 39 1246–1259. 10.1038/s41388-019-1061-6 31616062 PMC7299239

[B3] An Y. Bi F. You Y. Liu X. Yang Q. (2018). Development of a novel autophagy-related prognostic signature for serous ovarian cancer. *J. Cancer* 9 4058–4071. 10.7150/jca.25587 30410611 PMC6218776

[B4] Barker T. Fulde G. Moulton B. Nadauld L. D. Rhodes T. (2020). An elevated neutrophil-to-lymphocyte ratio associates with weight loss and cachexia in cancer. *Sci. Rep.* 10:7535. 10.1038/s41598-020-64282-z 32371869 PMC7200806

[B5] Broustas C. G. Duval A. J. Chaudhary K. R. Friedman R. A. Virk R. K. Lieberman H. B. (2020). Targeting MEK5 impairs nonhomologous end-joining repair and sensitizes prostate cancer to DNA damaging agents. *Oncogene* 39 2467–2477. 10.1038/s41388-020-1163-1 31980741 PMC7085449

[B6] Cancer Genome Atlas Research Network (2013). Comprehensive molecular characterization of clear cell renal cell carcinoma. *Nature* 499 43–49. 10.1038/nature12222 23792563 PMC3771322

[B7] Carril-Ajuria L. Santos M. Roldan-Romero J. M. Rodriguez-Antona C. de Velasco G. (2019). Prognostic and predictive value of PBRM1 in clear cell renal cell carcinoma. *Cancers* 12:16. 10.3390/cancers12010016 31861590 PMC7016957

[B8] Caruso J. A. Hunt K. K. Keyomarsi K. (2010). The neutrophil elastase inhibitor elafin triggers rb-mediated growth arrest and caspase-dependent apoptosis in breast cancer. *Cancer Res.* 70 7125–7136. 10.1158/0008-5472.CAN-10-1547 20823156 PMC2940941

[B9] Chandler B. C. Moubadder L. Ritter C. L. Liu M. Cameron M. Wilder-Romans K. (2020). TTK inhibition radiosensitizes basal-like breast cancer through impaired homologous recombination. *J. Clin. Invest.* 130 958–973. 10.1172/JCI130435 31961339 PMC6994133

[B10] Chatzinikolaou G. Karakasilioti I. Garinis G. A. (2014). DNA damage and innate immunity: links and trade-offs. *Trends Immunol.* 35 429–435. 10.1016/j.it.2014.06.003 25023467

[B11] Chen G. Zhou J. Chen J. Zhu J. Liu S. C. Ding X. F. (2019). VHL regulates NEK1 via both HIF-2alpha pathway and ubiquitin-proteasome pathway in renal cancer cell. *Biochem. Biophys. Res. Commun.* 509 797–802. 10.1016/j.bbrc.2019.01.001 30635121

[B12] Clayton E. A. Pujol T. A. McDonald J. F. Qiu P. (2020). Leveraging TCGA gene expression data to build predictive models for cancer drug response. *BMC Bioinform.* 21(Suppl. 14):364. 10.1186/s12859-020-03690-4 32998700 PMC7526215

[B13] Coffelt S. B. Kersten K. Doornebal C. W. Weiden J. Vrijland K. Hau C. S. (2015). IL-17-producing gammadelta T cells and neutrophils conspire to promote breast cancer metastasis. *Nature* 522 345–348. 10.1038/nature14282 25822788 PMC4475637

[B14] Deryugina E. I. Zajac E. Juncker-Jensen A. Kupriyanova T. A. Welter L. Quigley J. P. (2014). Tissue-infiltrating neutrophils constitute the major in vivo source of angiogenesis-inducing MMP-9 in the tumor microenvironment. *Neoplasia* 16 771–788. 10.1016/j.neo.2014.08.013 25379015 PMC4212255

[B15] Dong H. Strome S. E. Salomao D. R. Tamura H. Hirano F. Flies D. B. (2002). Tumor-associated B7-H1 promotes T-cell apoptosis: a potential mechanism of immune evasion. *Nat. Med.* 8 793–800. 10.1038/nm730 12091876

[B16] Feng H. Zhong L. Yang X. Wan Q. Pei X. Wang J. (2020). Development and validation of prognostic index based on autophagy-related genes in patient with head and neck squamous cell carcinoma. *Cell Death Discov.* 6:59. 10.1038/s41420-020-00294-y 32695478 PMC7360573

[B17] Galdiero M. R. Varricchi G. Loffredo S. Mantovani A. Marone G. (2018). Roles of neutrophils in cancer growth and progression. *J. Leukoc. Biol.* 103 457–464. 10.1002/JLB.3MR0717-292R 29345348

[B18] Garje R. An J. Greco A. Vaddepally R. K. Zakharia Y. (2020). The future of immunotherapy-based combination therapy in metastatic renal cell carcinoma. *Cancers* 12:143. 10.3390/cancers12010143 31936065 PMC7017064

[B19] Garsed D. W. Alsop K. Fereday S. Emmanuel C. Kennedy C. J. Etemadmoghadam D. (2018). Homologous recombination DNA repair pathway disruption and retinoblastoma protein loss are associated with exceptional survival in high-grade serous ovarian cancer. *Clin. Cancer Res.* 24 569–580. 10.1158/1078-0432.CCR-17-1621 29061645

[B20] Ged Y. Chaim J. L. DiNatale R. G. Knezevic A. Kotecha R. R. Carlo M. I. (2020). DNA damage repair pathway alterations in metastatic clear cell renal cell carcinoma and implications on systemic therapy. *J. Immunother. Cancer* 8:e000230. 10.1136/jitc-2019-000230 32571992 PMC7311069

[B21] Hu D. Jiang L. Luo S. Zhao X. Hu H. Zhao G. (2020). Development of an autophagy-related gene expression signature for prognosis prediction in prostate cancer patients. *J. Transl. Med.* 18:160 10.1186/s12967-020-02323-x PMC7137440 32264916

[B22] Huang Q. Sun Y. Zhai W. Ma X. Shen D. Du S. (2020). Androgen receptor modulates metastatic routes of VHL wild-type clear cell renal cell carcinoma in an oxygen-dependent manner. *Oncogene* 39 6677–6691. 10.1038/s41388-020-01455-0 32943729

[B23] Ina S. Hirono S. Noda T. Yamaue H. (2010). Identifying molecular markers for chemosensitivity to gemcitabine in pancreatic cancer: increased expression of interferon-stimulated gene 15 kd is associated with intrinsic chemoresistance. *Pancreas* 39 473–485. 10.1097/MPA.0b013e3181c0decc 19959962

[B24] Jiang P. Sun W. Shen N. Huang X. Fu S. (2020). Identification of a metabolism-related gene expression prognostic model in endometrial carcinoma patients. *BMC Cancer* 20:864. 10.1186/s12885-020-07345-8 32894095 PMC7487491

[B25] Jiang Y. Z. Liu Y. Xiao Y. Hu X. Jiang L. Zuo W. J. (2020). Molecular subtyping and genomic profiling expand precision medicine in refractory metastatic triple-negative breast cancer: the FUTURE trial. *Cell Res.* 1–9. 10.1038/s41422-020-0375-9 32719455 PMC8027015

[B26] Jiang Y. Z. Ma D. Suo C. Shi J. Xue M. Hu X. (2019). Genomic and transcriptomic landscape of triple-negative breast cancers: subtypes and treatment strategies. *Cancer Cell* 35 428–440.e5. 10.1016/j.ccell.2019.02.001 30853353

[B27] Jiao S. Xia W. Yamaguchi H. Wei Y. Chen M. K. Hsu J. M. (2017). PARP inhibitor upregulates PD-L1 expression and enhances cancer-associated immunosuppression. *Clin. Cancer Res.* 23 3711–3720. 10.1158/1078-0432.CCR-16-3215 28167507 PMC5511572

[B28] Jungnickel C. Schmidt L. H. Bittigkoffer L. Wolf L. Wolf A. Ritzmann F. (2017). IL-17C mediates the recruitment of tumor-associated neutrophils and lung tumor growth. *Oncogene* 36 4182–4190. 10.1038/onc.2017.28 28346430

[B29] Liu Y. Wu J. Huang W. Weng S. Wang B. Chen Y. (2020). Development and validation of a hypoxia-immune-based microenvironment gene signature for risk stratification in gastric cancer. *J. Transl. Med.* 18:201. 10.1186/s12967-020-02366-0 32410620 PMC7226948

[B30] Lok B. H. Gardner E. E. Schneeberger V. E. Ni A. Desmeules P. Rekhtman N. (2017). PARP inhibitor activity correlates with SLFN11 expression and demonstrates synergy with temozolomide in small cell lung cancer. *Clin. Cancer Res.* 23 523–535. 10.1158/1078-0432.CCR-16-1040 27440269 PMC5241177

[B31] Mauri G. Arena S. Siena S. Bardelli A. Sartore-Bianchi A. (2020). The DNA damage response pathway as a land of therapeutic opportunities for colorectal cancer. *Ann. Oncol.* 31 1135–1147. 10.1016/j.annonc.2020.05.027 32512040

[B32] Michaeli J. Shaul M. E. Mishalian I. Hovav A. H. Levy L. Zolotriov L. (2017). Tumor-associated neutrophils induce apoptosis of non-activated CD8 T-cells in a TNFalpha and NO-dependent mechanism, promoting a tumor-supportive environment. *Oncoimmunology* 6:e1356965. 10.1080/2162402X.2017.1356965 29147615 PMC5674962

[B33] Mishalian I. Bayuh R. Levy L. Zolotarov L. Michaeli J. Fridlender Z. G. (2013). Tumor-associated neutrophils (TAN) develop pro-tumorigenic properties during tumor progression. *Cancer Immunol. Immunother.* 62 1745–1756. 10.1007/s00262-013-1476-9 24092389 PMC11028422

[B34] Mollinedo F. (2019). Neutrophil degranulation, plasticity, and cancer metastasis. *Trends Immunol.* 40 228–242. 10.1016/j.it.2019.01.006 30777721

[B35] Motzer R. J. Escudier B. George S. Hammers H. J. Srinivas S. Tykodi S. S. (2020). Nivolumab versus everolimus in patients with advanced renal cell carcinoma: updated results with long-term follow-up of the randomized, open-label, phase 3 CheckMate 025 trial. *Cancer* 126 4156–4167. 10.1002/cncr.33033 32673417 PMC8415096

[B36] Motzer R. J. Escudier B. McDermott D. F. George S. Hammers H. J. Srinivas S. (2015). Nivolumab versus Everolimus in advanced renal-cell carcinoma. *N. Engl. J. Med.* 373 1803–1813. 10.1056/NEJMoa1510665 26406148 PMC5719487

[B37] Mu Y. Lou J. Srivastava M. Zhao B. Feng X. H. Liu T. (2016). SLFN11 inhibits checkpoint maintenance and homologous recombination repair. *EMBO Rep.* 17 94–109. 10.15252/embr.201540964 26658330 PMC4718411

[B38] Murai J. Feng Y. Yu G. K. Ru Y. Tang S. W. Shen Y. (2016). Resistance to PARP inhibitors by SLFN11 inactivation can be overcome by ATR inhibition. *Oncotarget* 7 76534–76550. 10.18632/oncotarget.12266 27708213 PMC5340226

[B39] Na J. C. Nagaya N. Rha K. H. Han W. K. Kim I. Y. (2019). DNA damage response pathway alteration in locally advanced clear-cell renal-cell carcinoma is associated with a poor outcome. *Clin. Genitourin. Cancer* 17 299–305.e1. 10.1016/j.clgc.2019.05.004 31204211

[B40] Powell D. R. Huttenlocher A. (2016). Neutrophils in the tumor microenvironment. *Trends Immunol.* 37 41–52. 10.1016/j.it.2015.11.008 26700397 PMC4707100

[B41] Sato H. Jeggo P. A. Shibata A. (2019). Regulation of programmed death-ligand 1 expression in response to DNA damage in cancer cells: implications for precision medicine. *Cancer Sci.* 110 3415–3423. 10.1111/cas.14197 31513320 PMC6824998

[B42] Sato H. Niimi A. Yasuhara T. Permata T. B. M. Hagiwara Y. Isono M. (2017). DNA double-strand break repair pathway regulates PD-L1 expression in cancer cells. *Nat. Commun.* 8:1751. 10.1038/s41467-017-01883-9 29170499 PMC5701012

[B43] Sen T. Rodriguez B. L. Chen L. Corte C. M. D. Morikawa N. Fujimoto J. (2019). Targeting DNA damage response promotes antitumor immunity through STING-mediated T-cell activation in small cell lung cancer. *Cancer Discov.* 9 646–661. 10.1158/2159-8290.CD-18-1020 30777870 PMC6563834

[B44] Shen M. Hu P. Donskov F. Wang G. Liu Q. Du J. (2014). Tumor-associated neutrophils as a new prognostic factor in cancer: a systematic review and meta-analysis. *PLoS One* 9:e98259. 10.1371/journal.pone.0098259 24906014 PMC4048155

[B45] Siegel R. L. Miller K. D. Jemal A. (2019). Cancer statistics, 2019. *CA Cancer J. Clin.* 69 7–34. 10.3322/caac.21551 30620402

[B46] Suh J. Jeong C. W. Choi S. Ku J. H. Kim H. H. Kim K. (2020). Sharing the initial experience of pan-cancer panel analysis in high-risk renal cell carcinoma in the Korean population. *BMC Urol.* 20:125 10.1186/s12894-020-00687-2 PMC7433120 32811483

[B47] Sun W. Zhang L. Luo M. Hu G. Mei Q. Liu D. (2016). Pretreatment hematologic markers as prognostic factors in patients with nasopharyngeal carcinoma: Neutrophil-lymphocyte ratio and platelet-lymphocyte ratio. *Head Neck* 38(Suppl. 1), E1332–E1340. 10.1002/hed.24224 26362911

[B48] Sun Y. Zhu D. Chen F. Qian M. Wei H. Chen W. (2016). SFRP2 augments WNT16B signaling to promote therapeutic resistance in the damaged tumor microenvironment. *Oncogene* 35 4321–4334. 10.1038/onc.2015.494 26751775 PMC4994019

[B49] Tecchio C. Scapini P. Pizzolo G. Cassatella M. A. (2013). On the cytokines produced by human neutrophils in tumors. *Semin. Cancer Biol.* 23 159–170. 10.1016/j.semcancer.2013.02.004 23410636

[B50] Templeton A. J. McNamara M. G. Seruga B. Vera-Badillo F. E. Aneja P. Ocana A. (2014). Prognostic role of neutrophil-to-lymphocyte ratio in solid tumors: a systematic review and meta-analysis. *J. Natl. Cancer Instit.* 106:dju124. 10.1093/jnci/dju124 24875653

[B51] Topalian S. L. Hodi F. S. Brahmer J. R. Gettinger S. N. Smith D. C. McDermott D. F. (2012). Safety, activity, and immune correlates of anti-PD-1 antibody in cancer. *N. Engl. J. Med.* 366 2443–2454. 10.1056/NEJMoa1200690 22658127 PMC3544539

[B52] Vidotto T. Nersesian S. Graham C. Siemens D. R. Koti M. (2019). DNA damage repair gene mutations and their association with tumor immune regulatory gene expression in muscle invasive bladder cancer subtypes. *J. Immunother. Cancer* 7:148. 10.1186/s40425-019-0619-8 31174611 PMC6556053

[B53] Wan B. Liu B. Huang Y. Yu G. Lv C. (2019a). Prognostic value of immune-related genes in clear cell renal cell carcinoma. *Aging* 11 11474–11489. 10.18632/aging.102548 31821170 PMC6932908

[B54] Wan B. Liu B. Yu G. Huang Y. Lv C. (2019b). Differentially expressed autophagy-related genes are potential prognostic and diagnostic biomarkers in clear-cell renal cell carcinoma. *Aging* 11 9025–9042. 10.18632/aging.102368 31626592 PMC6834403

[B55] Wang Q. Tan Y. Fang C. Zhou J. Wang Y. Zhao K. (2019). Single-cell RNA-seq reveals RAD51AP1 as a potent mediator of EGFRvIII in human glioblastomas. *Aging* 11 7707–7722. 10.18632/aging.102282 31532757 PMC6781999

[B56] Wang Y. Guo X. Bray M. J. Ding Z. Zhao Z. (2016). An integrative genomics approach for identifying novel functional consequences of PBRM1 truncated mutations in clear cell renal cell carcinoma (ccRCC). *BMC Genom.* 17(Suppl. 7):515. 10.1186/s12864-016-2906-9 27556922 PMC5001239

[B57] Wislez M. Antoine M. Rabbe N. Gounant V. Poulot V. Lavole A. (2007). Neutrophils promote aerogenous spread of lung adenocarcinoma with bronchioloalveolar carcinoma features. *Clin. Cancer Res.* 13 3518–3527. 10.1158/1078-0432.CCR-06-2558 17575214

[B58] Wu Y. Wang H. Qiao L. Jin X. Dong H. Wang Y. (2019). Silencing of RAD51AP1 suppresses epithelial-mesenchymal transition and metastasis in non-small cell lung cancer. *Thorac. Cancer* 10 1748–1763. 10.1111/1759-7714.13124 31317661 PMC6718026

[B59] Zeng D. Li M. Zhou R. Zhang J. Sun H. Shi M. (2019). Tumor microenvironment characterization in gastric cancer identifies prognostic and immunotherapeutically relevant gene signatures. *Cancer Immunol. Res.* 7 737–750. 10.1158/2326-6066.CIR-18-0436 30842092

[B60] Zhang L. Zhang Z. Yu Z. (2019). Identification of a novel glycolysis-related gene signature for predicting metastasis and survival in patients with lung adenocarcinoma. *J. Transl. Med.* 17:423. 10.1186/s12967-019-02173-2 31847905 PMC6916245

[B61] Zhou S. L. Zhou Z. J. Hu Z. Q. Huang X. W. Wang Z. Chen E. B. (2016). Tumor-associated neutrophils recruit macrophages and T-Regulatory cells to promote progression of hepatocellular carcinoma and resistance to Sorafenib. *Gastroenterology* 150 1646–1658.e17. 10.1053/j.gastro.2016.02.040 26924089

